# High-Resolution Diffusion-weighted Imaging Increases Prostate Cancer Visibility?

**DOI:** 10.1016/j.ebiom.2016.04.015

**Published:** 2016-04-16

**Authors:** Jurgen J. Fütterer

**Affiliations:** Department of Radiology and Nuclear Medicine (766), Radboud University Medical Centre Nijmegen, Geert Grooteplein 10, 6500HB Nijmegen, The Netherlands

Multiparametric MRI of the prostate has become a valuable imaging tool to detect clinically significant prostate cancer (Panebianco et al., 2015, Schoots et al., 2015). This tool consists of anatomical and functional imaging techniques. Currently, diffusion-weighted imaging is the most promising functional technique to detect and characterize prostate cancer. Despite its huge potential, it has some limitations that may hamper further implementation in the clinical practice. The signal-to-noise ratio is currently the major limitation. As a result, the highest in plane resolution, achievable with commercially available sequences, is in the range of 1.5 mm (Barentsz et al., 2012).

In *EBioMedicine*, Sharif-Afshar et al. present an important step forward to overcome the current limitations of diffusion-weighted imaging (Sharif-Afshar et al., 2016). This prospective pilot study included 17 men scheduled for prostate biopsy for active surveillance. They underwent multiparametric MRI of the prostate prior to the prostate biopsy. Additionally, the men underwent a high resolution three-dimensional multi-shot diffusion-weighted imaging sequence, which has advantages over two-dimensional single-shot echo-planer imaging. These include markedly reduced geometric distortion, minimization of susceptibility-related artifacts, and higher achievable spatial resolution. This multishot technique allows differentiation of signal and noise, further improving resolution. The reported in plane resolution is 0.9 × 0.9 × 3.5 mm^3^, which is improved by a factor 4.4 to 5.4. This resulted in a significantly higher area under the ROC for the high resolution diffusion-weighted MRI compared to the standard clinical diffusion-weighted imaging. Subsequently, the new technique identified significantly more biopsy-proven lesions than standard diffusion MRI.

The investigated functional imaging technique has great potentials. Diffusion-weighted MRI may provide an aid to establish the aggressiveness of prostate cancer and help predict those tumors most likely to progress rapidly. Prostate biopsies frequently underestimate the ‘true’ Gleason score of tumors found with transrectal ultrasound compared to radical prostatectomy (ranging from 30 to 50%, depending on initial Gleason score) (Pinthus et al., 2006, Corcoran et al., 2011). This high-resolution technique may further improve lesion characterization and ultimately be used to perform MRI diffusion-weighted imaging-targeted biopsy.

A question that arises is whether this higher spatial resolution will improve patient selection for active surveillance. Particular in this patient group, it is difficult to find low-grade lesions with diffusion-weighted imaging. Contrast-enhanced MRI is more helpful in finding those lesions. On the other hand, the proposed technique may be used to exclude males from the active surveillance program.

To conclude, high-resolution diffusion-weighted imaging of the prostate has the potential to further improve lesion characterization and visibility. It has paved the way for more accurate MRI-targeted biopsy.

## Disclosure

The author declared no conflicts of interest.
